# Nanocrystalline TiO_2_/Carbon/Sulfur Composite Cathodes for Lithium–Sulfur Battery

**DOI:** 10.3390/nano11020541

**Published:** 2021-02-20

**Authors:** Markéta Zukalová, Monika Vinarčíková, Milan Bouša, Ladislav Kavan

**Affiliations:** J. Heyrovský Institute of Physical Chemistry, Czech Acad. Sci., Dolejškova 3, CZ-18223 Prague 8, Czech Republic; monika.vinarcikova@jh-inst.cas.cz (M.V.); milan.bousa@jh-inst.cas.cz (M.B.); ladislav.kavan@jh-inst.cas.cz (L.K.)

**Keywords:** lithium–sulfur, titanium oxide, active carbon, electrochemical carbon, mesoporous carbon, battery, surface area

## Abstract

This paper evaluates the influence of the morphology, surface area, and surface modification of carbonaceous additives on the performance of the corresponding cathode in a lithium–sulfur battery. The structure of sulfur composite cathodes with mesoporous carbon, activated carbon, and electrochemical carbon is studied by X-ray diffraction, nitrogen adsorption measurements, and Raman spectroscopy. The sulfur cathode containing electrochemical carbon with the specific surface area of 1606.6 m^2^ g^−1^ exhibits the best electrochemical performance and provides a charge capacity of almost 650 mAh g^−1^ in cyclic voltammetry at a 0.1 mV s^−1^ scan rate and up to 1300 mAh g^−1^ in galvanostatic chronopotentiometry at a 0.1 C rate. This excellent electrochemical behavior is ascribed to the high dispersity of electrochemical carbon, enabling a perfect encapsulation of sulfur. The surface modification of carbonaceous additives by TiO_2_ has a positive effect on the electrochemical performance of sulfur composites with mesoporous and activated carbons, but it causes a loss of dispersity and a consequent decrease of the charge capacity of the sulfur composite with electrochemical carbon. The composite of sulfur with TiO_2_-modified activated carbon exhibited the charge capacity of 393 mAh g^−1^ in cyclic voltammetry and up to 493 mAh g^−1^ in galvanostatic chronopotentiometry. The presence of an additional Sigracell carbon felt interlayer further improves the electrochemical performance of cells with activated carbon, electrochemical carbon, and nanocrystalline TiO_2_-modified activated carbon. This positive effect is most pronounced in the case of activated carbon modified by nanocrystalline TiO_2_. However, it is not boosted by additional coverage by TiO_2_ or SnO_2_, which is probably due to the blocking of pores.

## 1. Introduction

As a result of the worldwide increasing demand for high energy and cost-effective energy storage technologies and the limited charge storage capacity of the current Li-ion batteries, the lithium sulfur (Li–S) battery is attracting attention from research teams all over the world. Considering the overall reaction S_8_ + 16 Li = 8 Li_2_S taking place in a cell with an Li metal anode and sulfur cathode, this system provides the theoretical specific capacity of 1675 Ah kg^−1^ and energy density of 2600 Wh kg^−1^. These values are unbeatable by any type of traditional Li-ion batteries. However, despite the effort of researchers, the lithium–sulfur battery represents still a great scientific challenge. The main problems are its short cycle life, low charging efficiency, poor safety, and high self-discharge rate [1]. The formation of soluble reduction intermediates (lithium polysulfides, Li_2_S_n_, further abbreviated PS) on the cathode and their subsequent diffusion to the anode causes the so-called shuttle effect, resulting in low Coulombic efficiency for charging and a fast self-discharge rate [1]. Nevertheless, the formation of soluble lithium polysulfides is essential for the operation of an Li–S battery. Since sulfur is non-conductive, the cathode is prepared as a composite of sulfur with a conductive additive. The most frequently used conductive additives are porous carbons of different origin and morphology [2,3,4,5,6,7], carbon nanotubes [2], graphene oxide [2,8], hollow carbon spheres or nanofibers [7], and their combination in a form of hierarchical materials [7,8,9,10]. They provide both high electronic conductivity and enough space to accommodate volume changes during charging and discharging. On a carbon surface, sulfur is reduced to soluble PS, which diffuse into the electrolyte solution and leave remaining sulfur exposed to the conductive carbon [1], where it is reduced. Thus, the dissolving of PS on the cathode pushes the reaction forward, but their diffusion to the anode and parasitic reactions with electrolyte solvents and Li anode worsen considerably the Coulombic efficiency, increase the self-discharge rate, and decrease the cell capacity [1]. Suppression of the above-mentioned issues consists in a confinement of PS diffusion to the cathode compartment, which can be achieved by an additional barrier layer between the cathode and the separator [11], appropriate separator modification [12], conductive additive structure engineering [3,6,8,10,13,14], or carbon additive surface functionalization [2,15,16,17,18,19]. Since the operation of the Li–S battery represents a complex problem involving both single- and two-phase processes, optimization of its performance requires a multifaceted approach as well. The confinement of sulfur in a carbonaceous additive with high surface area and porosity facilitates its complete conversion to PS. Their subsequent diffusion can be suppressed both by the porous structure of carbon and by the modification of its surface by doping or functionalization [13,15,16,17,18,20]. Oxidic surface groups adsorb PS more efficiently than bare carbon. The PS trapping ability of different metal oxides, both electrochemically inactive and active, is extensively studied [1,7,21]. Considering both environmental and economical aspects, TiO_2_ adsorbed on carbon surface appears as a promising candidate. Titanium oxide is non-toxic, abundant, and cheap. As a result of its versatile applicability, there are numerous publications dealing with syntheses producing TiO_2_ with different crystal modification, particle size, and morphology [22]. The strong adsorption of PS on TiO_2_ is the main reason for the improved performance of the carbon–TiO_2_–sulfur composite cathode in the Li–S battery [15,16,17]. The carbon/TiO_2_ composite provides a platform on which PS adsorption and cross-surface diffusion occur in series. Both processes should be fast enough to lead the lithium polysulfides toward the carbon surface, where the electrochemical reaction takes place [7]. Hence, the surface properties of metal oxide are decisive for the subsequent diffusion of PS, and high surface area metal oxides are favorable for this application [7]. Since we studied extensively the preparation and properties of nanocrystalline TiO_2_ [23,24], it was challenging to test the PS trapping performance of sol–gel made nanocrystalline TiO_2_ anchored on a carbon–sulfur composite cathode. The optimum concept of cathode composite for the Li–S battery should be designed as high surface area porous carbon decorated with small metal oxide particles in a concentration of 5–20 wt% covered with sulfur. Finally, an additional ionically conductive porous interlayer between the cathode and separator hinders PS diffusion to the anode compartment and thus improves the cell stability and Coulombic efficiency as well as suppresses the self-discharge.

Here, we demonstrate the influence of surface area and porosity of carbonaceous additive on the electrochemical performance of an Li–S battery. We compare the performance of composite sulfur cathodes containing mesoporous carbon, commercial activated carbon, and our home-made electrochemical carbon [25]. In addition, we prepared nanocrystalline TiO_2_/carbon/sulfur composite cathodes and found that the presence of nanocrystalline TiO_2_ on a surface of activated carbon further improves its performance. An additional Sigracell interlayer impeding PS diffusion to the separator and anode enhances the long-term stability of the cell.

## 2. Materials and Methods

### 2.1. Preparation of Materials

Carbonaceous additives (mesoporous carbon, MP, Aldrich, activated carbon, AC, Aldrich, electrochemical carbon, EC, the latter prepared by a procedure described in Ref. [25]) was mixed with sulfur (Aldrich, St. Louis, MO, USA) in a mass ratio 1:3. The mixture was mortared and then treated at 155 °C for 15 h under Ar atmosphere in a Teflon container. The product was again mortared and mixed with conductive carbon black C65 (Timcal, dried at 230 °C in vacuum) and polyvinylpyrrolidone (PVP, Aldrich). The mass ratio of the carbon/sulfur composite to C65 and PVP was 7:2:1. (The concentration of sulfur in the final electrode material was 52.5 wt%). Then, the mixture was suspended in 1-methyl 2-pyrrolidinone (NMP, 99.7%, Aldrich) to a consistence of viscous paste and coated by doctor-blading on Al foil. After drying in air at ambient temperature and at 100 °C in vacuum overnight, the coated Al foil was cut into disc electrodes of 15 mm in diameter. The areal sulfur loading was adjusted to 1–2 mg cm^−2^. The electrodes were stored in a glove box with Ar atmosphere. 

A TiO_2_–carbon composite was prepared by impregnation of the dried carbonaceous additive on a Büchner funnel under suction. The material was first soaked with 2-propanol (p.a., Aldrich) and then with Ti-isopropoxide (97%, Aldrich). After drying at ambient temperature in air overnight, it was treated with sulfur as described above. The concentration of TiO_2_ in the TiO_2_–carbon composite was determined by gravimetric analysis. Both the dried composite and pure carbon were annealed at 600 °C in air for 2 h. TiO_2_ content (15.9% for MP + TiO_2_, 15.6% for AC + TiO_2_, and 14.7% for EC + TiO_2_) was calculated as a mass difference of the composite before and after annealing corrected with respect to the ash residue in particular carbon. The concentration of TiO_2_ in the final MP/AC/EC + TiO_2_ + S composite was ~3%.

A Sigracell (SGL Carbon) interlayer was dried at 100 °C in vacuum overnight and cut into discs of 15 mm in diameter. The mass of a typical disc was 61 mg, and its thickness 1.16 mm. In some cases, the surface of the interlayer was covered by a 50 nm film of TiO_2_ or SnO_2_ that was fabricated by atomic layer deposition (ALD).

The electrolyte consisted of 1.0 M lithium bis-(trifluoromethanesulfonyl) imide (LiTFSI) dissolved in a mixture of 1,3-dioxolane and 1,2-dimethoxyethane (1:1 by volume) with 1.0 wt% LiNO_3_ as an electrolyte-additive. LiTFSI was dried in vacuum at 130 °C overnight. Electrolytes, solvents, and redox-active molecules were of standard quality (p.a. or electrochemical grade) purchased from Aldrich or Merck and used as received. The standard amount of electrolyte in a cell was 20 μL.

### 2.2. Methods

Atomic layer deposition employed the R200 (Picosun, Finland) reactor. The TiO_2_ films were prepared at 150 °C using tetrakis(dimethylamino)titanium (99.99%, Aldrich), and the auxiliary reactant was water. SnO_2_ films were prepared at 118 °C using tetrakis-(dimethylamino)tin(IV) (99.99%-Sn, Strem Chemicals, Inc., Newburyport, MA, USA) and water. 

X-ray diffraction was measured on the Bruker D8 Advance diffractometer using CuKα radiation. Adsorption measurements of nitrogen were carried out with an ASAP 2020 apparatus (Micromeritics) at 77 K. All samples were degassed prior to analysis at 250 °C in vacuum. The surface area was determined by BET (Brunauer, Emmett, Teller) equation. A semiempirical t-plot method was employed for calculation of the micropores’ area and volume. The FE SEM S-4800 microscope (Hitachi, Tokyo, Japan) equipped with the energy-dispersive X-ray spectroscope (EDX, Noran EDX system) served for examination of scanning electron microscopy (SEM) images and surface elemental analysis. Raman spectra were measured on MicroRaman system (LabRAM HR spectrometer, Horiba Jobin-Yvon) with an Olympus BX microscope. The spectra were excited by an He-Ne laser (633 nm). Electrochemical measurements were carried out with Autolab 302N apparatus (Metrohm) controlled by Nova and Nova Battery SW in a Swagelok-type cell with Li-metal anode and polypropylene separator (Targray) in an Ar-filled glove box. Galvanostatic chronopotentiometry was measured in the 2032 coin-type test cells by the Neware Battery Testing System controlled by BTS 7.6 SW.

## 3. Results and Discussion

### 3.1. Materials Characterization

The structure of the prepared composite samples was analyzed by X-ray diffraction. Owing to amorphous nature of all carbonaceous additives, only the diffraction features of crystalline orthorhombic sulfur (pattern JCPDS: #08-0247) are dominating the diffractograms (data not shown).

**Figure 1:**
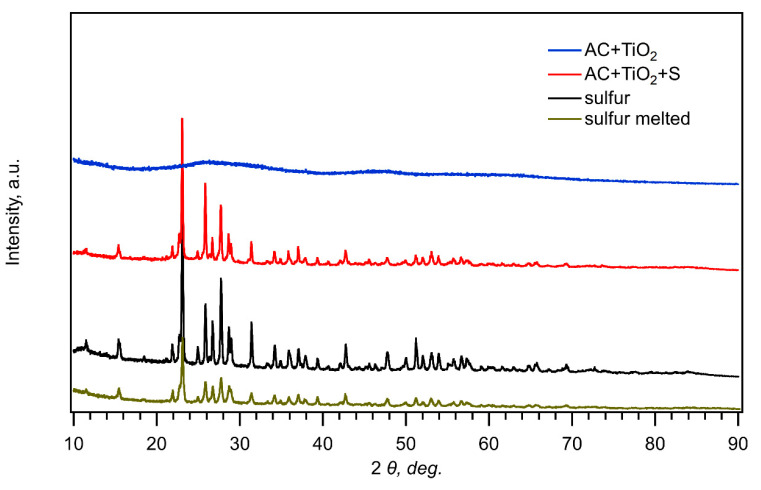
X-ray diffractograms of pure sulfur, melted sulfur, its composite with AC + TiO_2_, and pure AC + TiO_2_.

Figure 1 presents comparison of X-ray diffractograms of pure sulfur, pure sulfur recrystallized after melting at 155 °C, its composite with nanocrystalline TiO_2_ and activated carbon (AC + TiO_2_ + S), and nanocrystalline TiO_2_ activated carbon without sulfur (AC + TiO_2_). As the melting temperature of sulfur is 115 °C, the used synthesis of composite materials (see Section 2) inherently includes the interaction of melted sulfur with carbon. Figure 1 confirms that the diffraction peaks of orthorhombic sulfur (JCPDS: # 08-0247) are dominating the diffractograms in all cases, similarly to the earlier study of composites with mesoporous carbon [26]. Titania fabricated by the hydrolysis of Ti-isopropoxide is expected to exhibit the anatase structure [26], yet the main diagnostic peak of TiO_2_ anatase at 25.2 deg (2*θ*) is completely overlapped by the strong peaks of sulfur, which is probably due to the smaller concentration and low crystallinity of TiO_2_ in the composite (Figure 1, blue and red curves).

**Figure 2:**
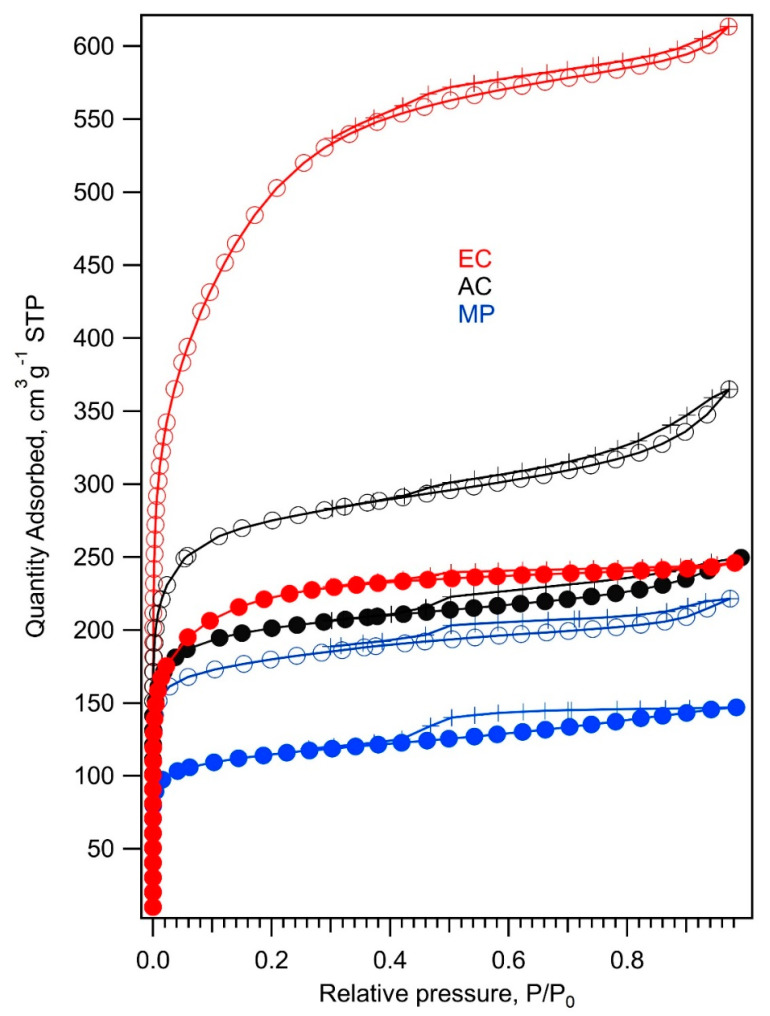
Nitrogen adsorption/desorption isotherms at 77 K of (from top to bottom) electrochemical carbon (EC, red line), commercial activated carbon (AC, black line) and mesoporous carbon (MP, blue line). The adsorption branch of the isotherm is marked with open circles, while the desorption branch is marked with crosses. Adsorption isotherms of carbon composites with TiO_2_ are depicted with full circles and lines of the same color as isotherms of corresponding pure carbon.

The reduction of sulfur takes place exclusively on the carbon surface [1]; hence, the surface area and porous structures of carbonaceous additives constitute the key factor for the electrochemical performance of the resulting composites with sulfur. Their structural parameters were investigated in detail by N_2_ adsorption isotherms. Figure 2 shows nitrogen adsorption isotherms at 77 K measured on mesoporous carbon (MP), commercial activated carbon (AC), electrochemical carbon (EC), and their composites with TiO_2_. A steep increase of the quantity adsorbed at low relative pressures >0.05 evidences the presence of micropores in all three carbonaceous samples [27]. Hysteresis occurring at relative pressures of 0.4 is caused by the capillary condensation of adsorbate (N_2_) in mesopores [27]. The values of BET surface areas, t-plot micropore areas, t-plot external surface areas, and t-plot micropore volumes are listed in Table 1. *Smic* and *Sext* were calculated using the t-plot method [28]. In this approach, the nitrogen adsorption isotherm is converted to a plot of adsorbed volume vs. the average thickness of the adsorbed layer determined by using a non-porous standard reference material of the same chemical composition [28]. For non-porous materials, the experimental points should then fall on a straight line through the origin [28]. The slope of this extrapolated line gives the specific surface area of the material. For microporous materials, the extrapolated line crosses the vertical axis at positive values. In this case, the slope of the extrapolated line determines the external surface area *Sext* of the material. Then, the surface area of micropores is calculated as a difference between *S_BET_* and *Sext*. 

**Figure 3:**
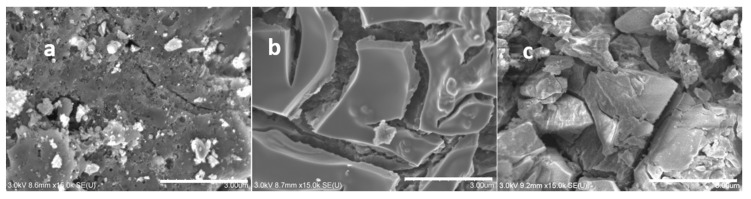
Scanning electron microscopy images of the (**a**) AC, (**b**) AC + TiO_2_, and (**c**) AC+ TiO_2_ + S samples. Scale bars 3 μm.

**Figure 4:**
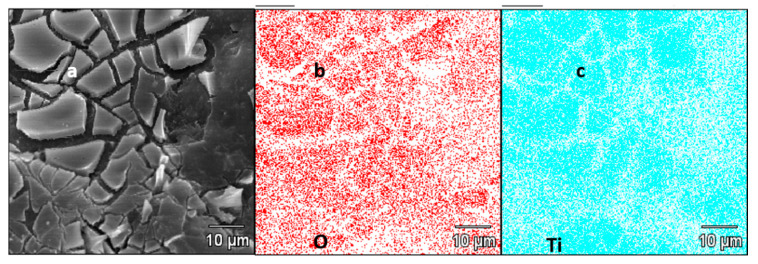
(**a**) SEM image of the AC + TiO_2_ sample and its (**b**) EDX concentration map of O and (**c**) EDX concentration map of Ti.

Figure 2 and Table 1 show that the MP and EC samples have almost the same volume of micropores, but the adsorption capacity of the EC sample is much higher due to its large external surface area (small particle size). The AC sample exhibits the largest volume of micropores, but its external surface area is just 30% larger than that of the MP sample. Both the porosity and large external surface area of carbonaceous additive are supposed to be beneficial for the electrochemical performance of the composites with sulfur. The large external surface of conductive additive enables a complete conversion of sulfur into PS, whereas the porous structure of carbon hinders the diffusion of the dissolved PS to the anode part of a cell. The nitrogen adsorption isotherms of carbonaceous composites with TiO_2_ maintain the shape of the corresponding carbon isotherms; nevertheless, they indicate a decrease of surface area by about 25–35% in the case of MP and AC samples due to partial coverage of the surface and pores by TiO_2_ (Figure 3 and Figure 4). The drop of surface area caused by the modification of carbon surface by TiO_2_ is most pronounced for the EC sample. Here, the TiO_2_ treatment results in a 60% decrease of the S_BET_ surface area. Table 1 and Figure 2 confirm that nanocrystalline TiO_2_ is located mostly on the external surface of the EC + TiO_2_ sample, whereas the micropores’ surface area of the original EC sample remained almost unchanged (differences in the t-plot micropore area of the EC samples and EC + TiO_2_ area are negligible within experimental error). This effect is expected, since nanocrystalline TiO_2_ cannot be accommodated in micropores narrower than 2 nm. 

Typical scanning electron microscopy images of the AC, AC + TiO_2_, and AC + TiO_2_ + S samples are presented in Figure 3.

The cracked TiO_2_ layer on the surface of AC is clearly visible in Figure 3b. Figure 3c further shows sulfur crystals covering the surface of the AC + TiO_2_ + S composite after the melting diffusion process (see the Experimental Section for details). Figure 4 depicts an SEM image of the sample AC + TiO_2_(a) and corresponding EDX concentration maps of O(b) and Ti(c) of the same focused area. The cracks and boundaries in Figure 4a are observable both in the concentration map of O and Ti in Figure 4b,c, which evidences the presence of pure TiO_2_ in the upper layer. The quantitative analysis of TiO_2_ content was carried out gravimetrically after thermal mineralization (see the Experimental Section). Specifically, our AC + TiO_2_ composite contained 15.6 wt% of TiO_2_.

**Figure 5:**
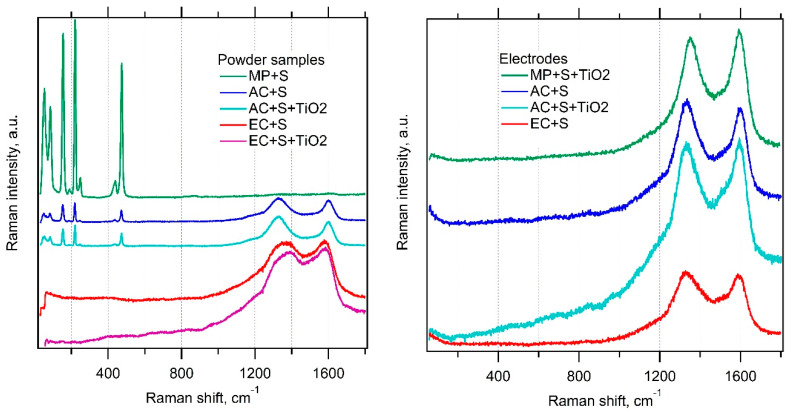
Raman spectra of the investigated materials in the form of starting powders (**left chart**) and complete electrodes in thin films at Al support (**right chart**). The spectra are offset for clarity, but the scale of intensity is the same in each chart.

Since X-ray analysis of composites confirmed convincingly just the presence of sulfur, Raman spectroscopy was employed as a complementary technique to analyze also the host materials in our composites. This method is known to be suitable not only for the investigation of electrode materials but also for the in situ spectroelectrochemical analysis of PS [26,29,30]. Figure 5 shows the corresponding spectra of our composite materials and the final electrodes. Powder composites of carbon with sulfur (prepared as detailed in the Experimental Section) exhibit pronounced changes, which are highly specific for each parent carbonaceous material. The mesoporous carbon (MP) exhibits dominating features of sulfur in the region from 20 to 500 cm^−1^ but a very weak signal of carbon in the region from 1200 to 1700 cm^−1^ (D and G modes).

On the other hand, the electrochemical carbon (EC) is characterized by just the opposite: strong carbon modes and a negligible signal of sulfur. The activated carbon (AC) shows an intermediate spectrum with both sulfur- and carbon-related features having roughly comparable intensities. Closer inspection reveals small frequency shifts of the D and G modes. Our observation confirms the previous work [26], which reported on a significant attenuation of the Raman features of sulfur in the adsorbed state on mesoporous carbon. While the cited work investigated solely the MP, here we extend this study by the investigation of two other carbon hosts with larger surface area. 

Obviously, the Raman intensities of sulfur/carbon strongly correlate with the respective surface area of the carbonaceous host. This confirms the hypothesis that the Raman signal of sulfur incorporated in the pores of carbon matrix is effectively attenuated. A similar observation was reported by Zeng et al. [31] in carbon nanocapsules loaded with sulfur from melt, who assigned the quenching of sulfur features to phonon confinement effects. The encapsulation of sulfur is virtually perfect in our electrochemical carbon (EC), exhibiting the highest specific surface area for sulfur anchoring. On the other hand, the mesoporous carbon (MP) with the smallest surface area still contains sulfur in the form of larger crystals, which are active for Raman scattering. (The limiting situation can be modeled by just mechanical mixing of both powders (S and MP) without melting [26]). The Raman signal of TiO_2_ is too weak to be traceable in all cases, which again matches the earlier study [26]. 

If the parent powder composites are included in thin-film electrodes with additives (PVP and C65 conductive carbon black), then the Raman spectrum is dominated solely by the D and G modes of carbon and neither sulfur nor TiO_2_ are traceable, even for the MP-based electrodes (Figure 5, right chart). In this case, the erasing of the Raman signal of sulfur cannot be caused by melting-driven nano-confinement, because the temperature during the electrode preparation did not exceed 100 °C (see the Experimental Section). Hence, the quenching of a sulfur signal in thin-film electrodes is ascribed to light absorption effects [26].

### 3.2. Electrochemical Measurements

**Figure 6:**
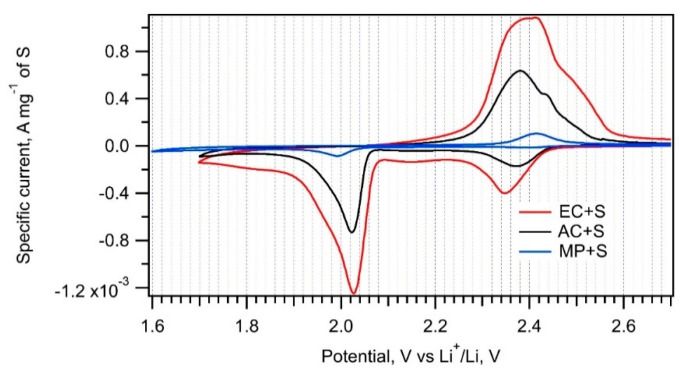
Cyclic voltammograms of the EC + S, AC + S, and MP + S samples recorded with a scan rate of 0.1 mV s^−1^.

**Figure 7:**
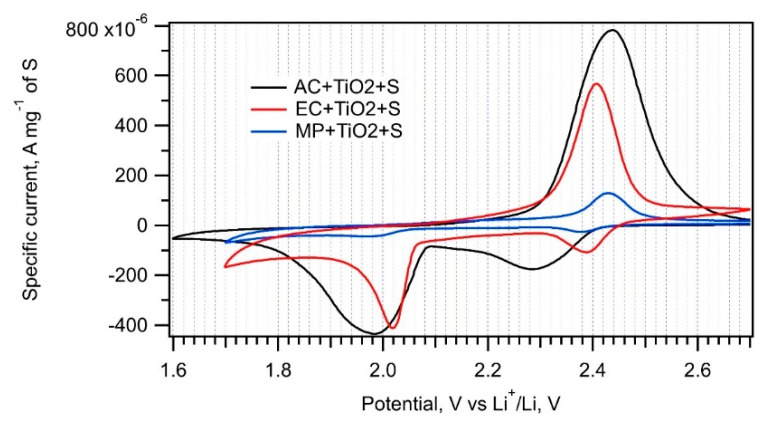
Cyclic voltammograms of the EC + TiO_2_ + S, AC + TiO_2_ + S and MP + TiO_2_ + S samples recorded with a scan rate of 0.1 mV s^−1^.

**Figure 8:**
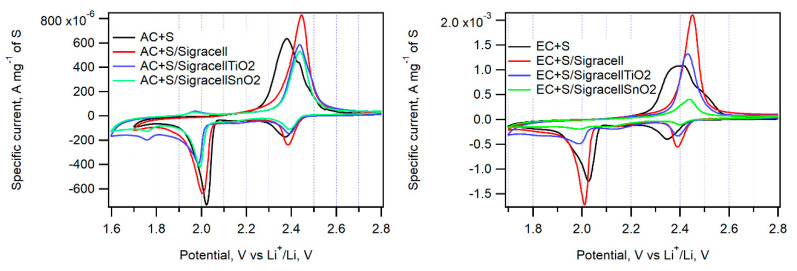
Cyclic voltammograms of EC + S and AC + S samples in cells without an interlayer (black curve) with the parent Sigracell interlayer (red curve) and with the Sigracell interlayer modified by metal oxide (TiO_2_-blue curve, SnO_2_-green curve) recorded with a scan rate of 0.1 mV s^−1^.

**Figure 9:**
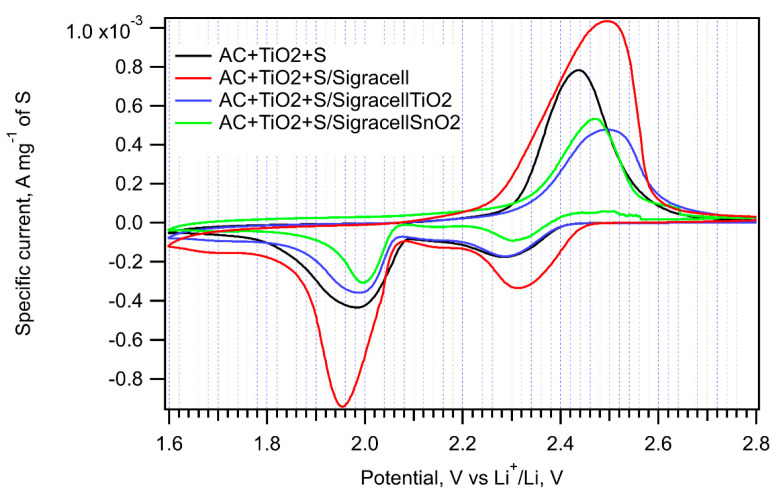
Cyclic voltammograms of the AC + TiO_2_ + S sample in cells without an interlayer (black curve), with the pure Sigracell interlayer (red curve) and with the modified Sigracell interlayer (TiO_2_—blue curve, SnO_2_—green curve) recorded with a scan rate of 0.1 mV s^−1^.

The electrochemical performance of MP, AC, and EC composites with sulfur was evaluated by cyclic voltammetry at the scan rate of 0.1 mV s^−1^ in a Swagelok-type cell (Figure 6). The safe potential window starts from the lower vertex potential of 1.7 V vs. Li^+^/Li, which is recommended as a prevention of reduction of LiNO_3_ electrolyte additive, severely affecting the reversibility of the sulfur cathode [1]. Yet, we show on one selected voltammogram (of MP + S) that there is still a negligible electrochemical activity even in the region of 1.6–1.7 V vs. Li^+^/Li. This negligible electrochemical activity allows using either of the lower vertex potentials (1.6 or 1.7) without significant influence on the integral voltammetric charge (cf. Figure 7, Figure 8 and Figure 9 below). 

Table 2 lists the charge capacities of all samples normalized to the mass of sulfur. The normalized cyclic voltammograms in Figure 6 show that the electrochemical performance of composites is significantly influenced by the particular carbonaceous additive. The charge capacities of the EC + S and AC + S samples (649 mAh g^−1^ and 319 mAh g^−1^, respectively) correlate very well with the surface areas of the parent carbons, i.e., EC and AC (Table 1). On the other hand, the charge capacity of the MP + S sample is negligible, despite just a 30% difference in the surface areas of MP and AC. Obviously, there are additional important factors influencing the sulfur reduction on a carbon–sulfur interface. To achieve maximum charge capacity, a carbonaceous additive must encase the particular sulfur component evenly and homogenously. Raman spectroscopy data (Figure 5, left chart) confirm that this condition is perfectly fulfilled for EC + S and partially for AC + S. In the case of MP + S, there is still a part of non-encapsulated sulfur in the sample, which cannot be reduced to PS [1]. This is a reason for the low charge capacity of the MP + S composite. Due to its extreme dispersity and large external surface area, the EC material represents an ideal additive for the sulfur cathode. 

The trapping of soluble PS by nanocrystalline metal oxides added to amorphous carbon is an efficient method to improve the cell’s capacity retention [1,32]. PS are adsorbed on a metal oxide surface or inside pores. Therefore, MP, AC, and EC were impregnated with nanocrystalline TiO_2_ prior to their interaction with sulfur melt. Cyclic voltammograms measured on the EC + TiO_2_ + S, AC + TiO_2_ + S, and MP + TiO_2_ + S samples are presented in Figure 7, and the corresponding charge capacities are shown in Table 2. 

The specific surface areas of the EC + TiO_2_, AC + TiO_2_, and MP + TiO_2_ samples exhibit an approximate 30% decrease compared to those of the parent carbons. This is caused by the partial surface and pore coverage by TiO_2_; nevertheless, this modification has a beneficial effect on the charge capacity of the AC + TiO_2_ + S and MP + TiO_2_ + S samples. Nanocrystalline TiO_2_ adsorbs PS more efficiently than pure carbon and could improve sulfur encapsulation or its adhesion to carbonaceous additive. The decreased charge capacity of EC + TiO_2_ + S compared to that of EC + S can be ascribed to a negative effect of solution-based TiO_2_ deposition onto fine-grained EC powder. As it was already discussed in the Section 3.1, EC dispersity, the main advantage of this material, is lost by this treatment, and the positive effect of nanocrystalline TiO_2_ on PS trapping is not sufficient to balance the decrease in active surface area. There is also a slightly higher peak-to-peak separation in the cyclic voltammograms of the composites modified by TiO_2_ due to their lower conductivity. Both cathodic and anodic peaks are shifted by ca. 60–70 mV to lower and higher potentials, respectively.

The incorporation of an additional interlayer in between the cathode and separator can efficiently hinder the diffusion of PS to the anode side of a cell. The optimum material for such a barrier layer must be electrochemically stable, porous, and able to adsorb PS. Here, we employed Sigracell carbon felt, which provides high porosity, electrochemical stability, and good elasticity. Sigracell carbon felt was used in cells with AC + S and EC + S cathodes as received, or it was covered with additional layer of TiO_2_ and SnO_2_ made by ALD. An additional thin oxide layer was expected to hinder further PS diffusion to the anode compartment due to TiO_2_’s ability to adsorb PS efficiently. A similar effect was observed for SnO_2_, too [33]. Figure 8 shows the cyclic voltammograms measured on cells with all three kinds of interlayers.

The pristine Sigracell interlayer significantly improves the charge capacity of cells with AC + S, EC + S, and AC + TiO_2_ + S cathodes. Its porous structure probably helps retain PS in the cathode compartment of the cell. On the other hand, its coverage by TiO_2_ or SnO_2_ decreases the cell charge capacity, which is probably due to the partial blocking of pores in carbon felt by metal oxide. 

The peak-to-peak separation caused by the Sigracell interlayer is higher on both the cathodic and anodic side compared to a cell without a barrier. The most pronounced effect of the Sigracell interlayer was observed for the AC + TiO_2_ + S cathode (Figure 9 and Table 2). Interestingly, in this sample, both the positive effect of pure carbon felt and the negative effect of subsequent modification with TiO_2_ or SnO_2_ are clearly pronounced. This can be ascribed to the facilitated reduction of sulfur adsorbed on nanocrystalline TiO_2_ in contact with conductive carbon felt. In any case, the beneficial effect of carbon felt is completely lost by metal oxide modification.

**Figure 10:**
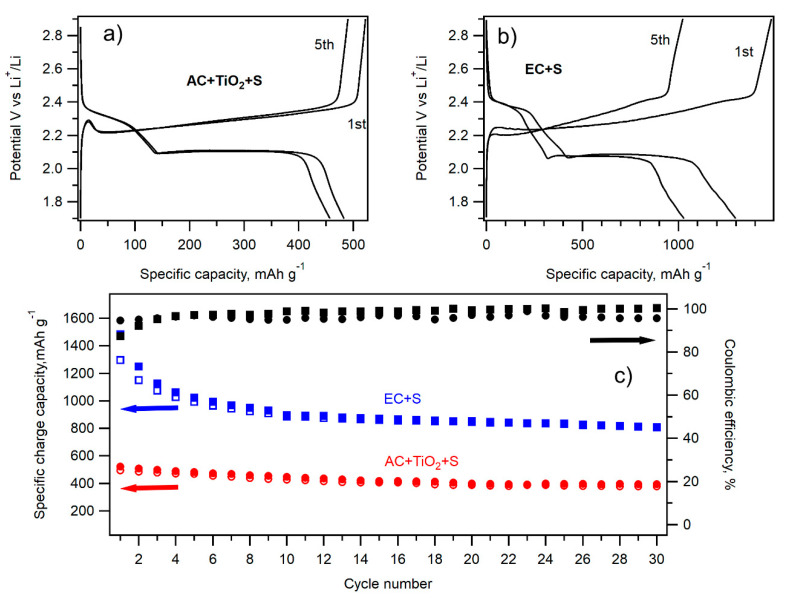
Charge–discharge curves of the 1st and 5th cycle of galvanostatic cycling at 0.1 C of the AC + TiO_2_ + S sample (**a**) and EC + S sample (**b**). (**c**) Galvanostatic chronopotentiometry data at 0.1 C of the AC + TiO_2_ + S (full red circles charge, open red circles discharge) and EC + S (full blue squares charge and open blue squares discharge) samples. The corresponding Coulombic efficiency values are depicted by full black circles (AC + TiO_2_ + S) and full black squares (EC + S).

Cycling performance of the most promising samples, i.e., EC + S and AC + TiO_2_ + S, was evaluated by galvanostatic chronopotentiometry at a 0.1 C charging/discharging rate. The charging/discharging curves of the 1st and 5th cycle are shown in Figure 10a,b. The electrochemical performance of both materials during 30 cycles of charging/discharging at the 0.1 C rate is depicted in Figure 10c. Whereas the specific charge/discharge capacity of the EC + S sample exhibits a pronounced decrease from the initial discharge capacity of 1297 mAh g^−1^ in the 1st cycle to 993 mAh g^−1^ in the 5th cycle, the discharge capacity of the AC + TiO_2_ + S sample is lower, but it drops by only 5% between the 1st and 5th cycle (493 and 468 mAh g^−1^). The impressive charge/discharge capacity of the EC + S sample is obviously an effect of the large surface area of this sample. The charge/discharge capacity of the sample AC + TiO_2_ + S attains values in the range of 400–500 mAh g^−1^. Contrary to the sample EC + S, the carbonaceous precursor AC of the AC + TiO_2_ + S sample is commercially available. The electrochemical performance of this sample can be further improved by the modification of its synthesis leading to the thinner layer or small isolated nanoislands of TiO_2_ on the surface of carbon. This is our research strategy for the near future.

## 4. Conclusions

The influence of the morphology and surface area of carbonaceous additives on the performance of the corresponding cathode in lithium–sulfur battery was evaluated. The structure of sulfur composite cathodes with mesoporous carbon, activated carbon, and electrochemical carbon was studied by X-ray diffraction, nitrogen adsorption measurements, and Raman spectroscopy. Their charge capacities were determined by cyclic voltammetry. The sulfur cathode containing electrochemical carbon with a surface area of 1606.6 m^2^ g^−1^ exhibited the best electrochemical performance and provided a charge capacity of almost 650 mAh g^−1^ in cyclic voltammetry at a 0.1 mV/s scan rate and up to 1300 mAh g^−1^ in galvanostatic chronopotentiometry at a 0.1 C rate. Based on Raman spectroscopy, this excellent electrochemical behavior was ascribed to high dispersity of electrochemical carbon enabling a perfect encapsulation of sulfur. The surface modification of carbonaceous additives by TiO_2_ has a positive effect on the electrochemical performance of sulfur composites with mesoporous and activated carbon, but it causes a loss of dispersity and a subsequent decrease of the charge capacity of the sulfur composite with electrochemical carbon. The composite of sulfur with TiO_2_-modified activated carbon exhibited the charge capacity of 393 mAh g^−1^ in cyclic voltammetry at a 0.1 mV s^−1^ scan rate and up to 493 mAh g^−1^ in galvanostatic chronopotentiometry at a 0.1 C rate. The presence of an additional Sigracell carbon felt interlayer further improves the electrochemical performance of cells with activated carbon, electrochemical carbon, and nanocrystalline TiO_2_-modified activated carbon. This positive effect is most pronounced in the case of nanocrystalline TiO_2_-modified activated carbon. However, it is not boosted by additional coverage of the interlayer by TiO_2_ or SnO_2_, which is probably due to the blocking of pores.

## Figures and Tables

**Table 1:** Structural parameters of carbonaceous additives.

Additive	*S_BET_*, m^2^ g^−1^	*Smic* (t-plot) m^2^ g^−1^	*Sext* (t-plot) m^2^ g^−1^	*Vmic* (t-plot) cm^3^ g^−1^
**MP**	521.5	372.4	149.1	0.21
**AC**	792.0	559.2	232.8	0.32
**EC**	1606.6	372.2	1234.4	0.22
**MP + TiO_2_**	340.3	226.8	113.5	0.13
**AC + TiO_2_**	574.2	429.7	144.5	0.25
**EC + TiO_2_**	659.6	381.6	278.0	0.22

**Table 2:** Charge capacities (C) of the samples and cells, calculated from cyclic voltammetry. Charge capacities in the 3rd, 4th, and 5th column were measured in cells with an additional Sigracell barrier (SG—pure Sigracell, SG + TiO_2_—Sigracell covered by 50 nm TiO_2_ layer by atomic layer deposition (ALD), SG + SnO_2_—Sigracell covered by 50 nm SnO_2_ layer by ALD).

Additive	C, mAh g^−1^	C, mAh g^−1^, SG	C, mAh g^−1^, SG + TiO_2_	C, mAh g^−1^, SG + SnO_2_
MP	72	-	-	-
AC	319	361	280	270
EC	649	765	521	278
MP + TiO_2_	95	-	-	-
AC + TiO_2_	393	660	305	271
EC + TiO_2_	417	-	-	-

## Data Availability

The data presented in this study are available on request from the corresponding author.
